# Case Report: Exceptional response to nivolumab plus cabozantinib in a patient with extrarenal clear cell renal cell carcinoma

**DOI:** 10.3389/fonc.2023.1271255

**Published:** 2023-10-04

**Authors:** Caroline S. Jansen, Yujin Choi, Sean T. Evans, Rachel Greenwald, Joseph A. Behnke, Caitlin Hartman, Haydn Kissick, Lara R. Harik, Mehmet Asim Bilen

**Affiliations:** ^1^ Department of Hematology and Medical Oncology, Emory University School of Medicine, Atlanta, GA, United States; ^2^ Winship Cancer Institute of Emory University, Atlanta, GA, United States; ^3^ Emory University School of Medicine, Atlanta, GA, United States; ^4^ Department of Medicine, Emory University School of Medicine, Atlanta, GA, United States; ^5^ Department of Urology, Emory University School of Medicine, Atlanta, GA, United States; ^6^ Department of Pathology and Laboratory Medicine, Emory University School of Medicine, Atlanta, GA, United States

**Keywords:** case report, extrarenal clear cell renal cell carcinoma, splenic renal cell carcinoma, nivolumab, cabozantinib, immunotherapy, targeted therapy, tyrosine kinase

## Abstract

Extrarenal clear cell renal cell carcinoma (eccRCC) is a rare type of RCC that arises in areas other than the kidney. Given its rarity, consensus guidelines for optimal treatment of eccRCC have not been established, and the literature is lacking any reports of patient response to systemic therapy and any reports of administration of immunotherapy to patients with ecRCC. Here, we present the case of a patient in their 60s with eccRCC arising in the spleen. The patient underwent splenic resection and then received systemic therapy, due to disease recurrence, with a combination of immunotherapy (IO) and tyrosine kinase inhibitor targeted therapy (VEGF-TKI). The patient had an excellent and durable response to this therapeutic regimen with minimal adverse effects, completing 2 years of therapy of nivolumab and cabozantinib. At the time of this report, the disease remains stable. This case demonstrates that combination therapy with IO+VEGF-TKI represents a reasonable and well-tolerated treatment option with activity in eccRCC and reveals interesting correlative data, including nests of stem-like CD8+T-cell infiltration in tumor tissue, which provide important biological context to this patient’s exceptional therapeutic response.

## Background

Approximately 400,000 cases of renal cell carcinoma are diagnosed each year, accounting for ~2.4% of new diagnoses of malignancy (1) and resulting in over 175,000 deaths globally (2). Clear cell renal cell carcinoma (ccRCC) is the most common type of renal cancer, representing 85% of all kidney cancer cases (3–5).

Primary renal cell carcinoma often presents asymptomatically, with only a minority of patients presenting with classic symptoms of hematuria, flank pain, or a palpable mass (5). As a result, as many as 25%–30% of patients present with metastatic disease (4). Patients with localized diseased are primarily treated with surgical resection with or without systemic therapy in the adjuvant therapy, and the 20%–40% of patients who recur after surgery (6), as well as patients presenting with metastatic disease, will also receive systemic therapy. The backbone of systemic therapy in the treatment of renal cell carcinoma is combination targeted therapy (i.e., VEGF tyrosine kinase inhibitors) and immunotherapy (i.e., immune checkpoint blockade) (7).

Extrarenal clear cell RCC (eccRCC) is an uncommon subtype of primary RCC rising in areas outside of the kidneys (3). EccRCC typically develops in abnormally located renal tissue or supranumerary kidneys (3, 8), but extremely rarely has been reported outside any known renal tissue or in the presence of normal bilateral kidneys (8–14). In these cases, it is hypothesized that the tumor may develop in tissues with shared embryologic origins with the kidney or in metanephric structures that aberrantly persist beyond gestation (8–10, 13). In the few reported cases of eccRCC without kidney involvement, most were treated with surgical resection without reports of recurrence during post-surgical surveillance (8–15). To our knowledge, there are only two reported cases in which systemic treatment was administered to the patient, with either tyrosine kinase inhibitors (TKIs) or chemotherapy. Notably and unfortunately, both of these cases provide limited information about effective treatment regimens in eccRCC, as in one case, the patient was offered palliative TKI but was lost to follow-up, and in the other, the patient rapidly expired due to aggressive progressive disease (16, 17). In the literature, there are no available reports of systemic treatment of eccRCC with a positive clinical response.

To our knowledge, this is the first case of long-term systemic treatment of eccRCC with any notable improvement in disease progression to be reported in literature. This is also the first reported case of a patient with metastatic eccRCC who had exceptional response to nivolumab and cabozantinib.

## Case presentation

**Figure 1:**
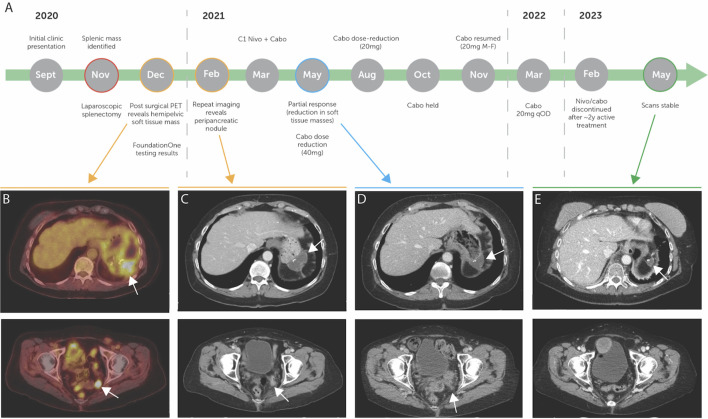
**(A)** Diagnosis and treatment timeline of the patient, with pre-treatment imaging highlighted in yellow, on treatment imaging highlighted in blue, and post-treatment imaging highlighted in green. **(B)** Pre-treatment PET scan showing FDG avid peripancreatic fluid collection and FGD avid pelvic soft tissue mass. **(C)** Pre-treatment CT scan showing peripancreatic nodule and redemonstrating pelvic soft tissue mass. **(D)** On-treatment CT scan showing partial response in peripancreatic and pelvic soft tissue masses. **(E)** Post-treatment CT scan demonstrating stable disease. **(B–E)** white arrows demonstrate highlighted areas of interest.

We report the case of a patient in their 60s with a history of Sjogren’s disease who presented to an outside hospital ambulatory clinic in September 2020 with left upper quadrant pain radiating to the shoulder, in the absence of any other clinical symptoms including weight loss or changes in appetite (**Figure 1A**). At that time, an abdominal ultrasound demonstrated a heterogeneous mass lesion extending from the splenic hilum, estimated to measure 7.4 × 7.3 × 7.4 cm in size. Subsequent abdominal CT scan demonstrated the presence of a 10.7 × 7.7 cm mass in the spleen, along with scattered splenic granulomatous lesions, without involvement of the pancreas. MRI of the abdomen redemonstrated the presence of an 8.4 × 7.6 cm mass in November 2020, and with a working diagnosis of carcinoma in the spleen with unclear primary, the patient underwent laparoscopic splenectomy in the same month.

Surgical pathology samples revealed an invasive high-grade neoplasm that was positive for pancytokeratin, cytokeratin 7, cytokeratin 5/6, CD10, and vimentin. Staining for cytokeratin 20, cytokeratin 19, Melan-A, S100, desmin, CD34, synaptophysin, chromogranin, TTF-1, estrogen receptor, progesterone receptor, and LCA yielded negative results. Based on this staining, outside pathologists felt that colorectal, breast, endometrioid, bladder, and lung primaries were unlikely and that staining suggested a high possibility of metastatic renal cell carcinoma. Postoperative PET scan revealed uptake in a nodule in the deep left pelvis, suspicious for recurrent disease, and postoperative fluid collection adjacent to the pancreatic tail and fundus of the stomach (**Figure 1B**).

**Figure 2:**
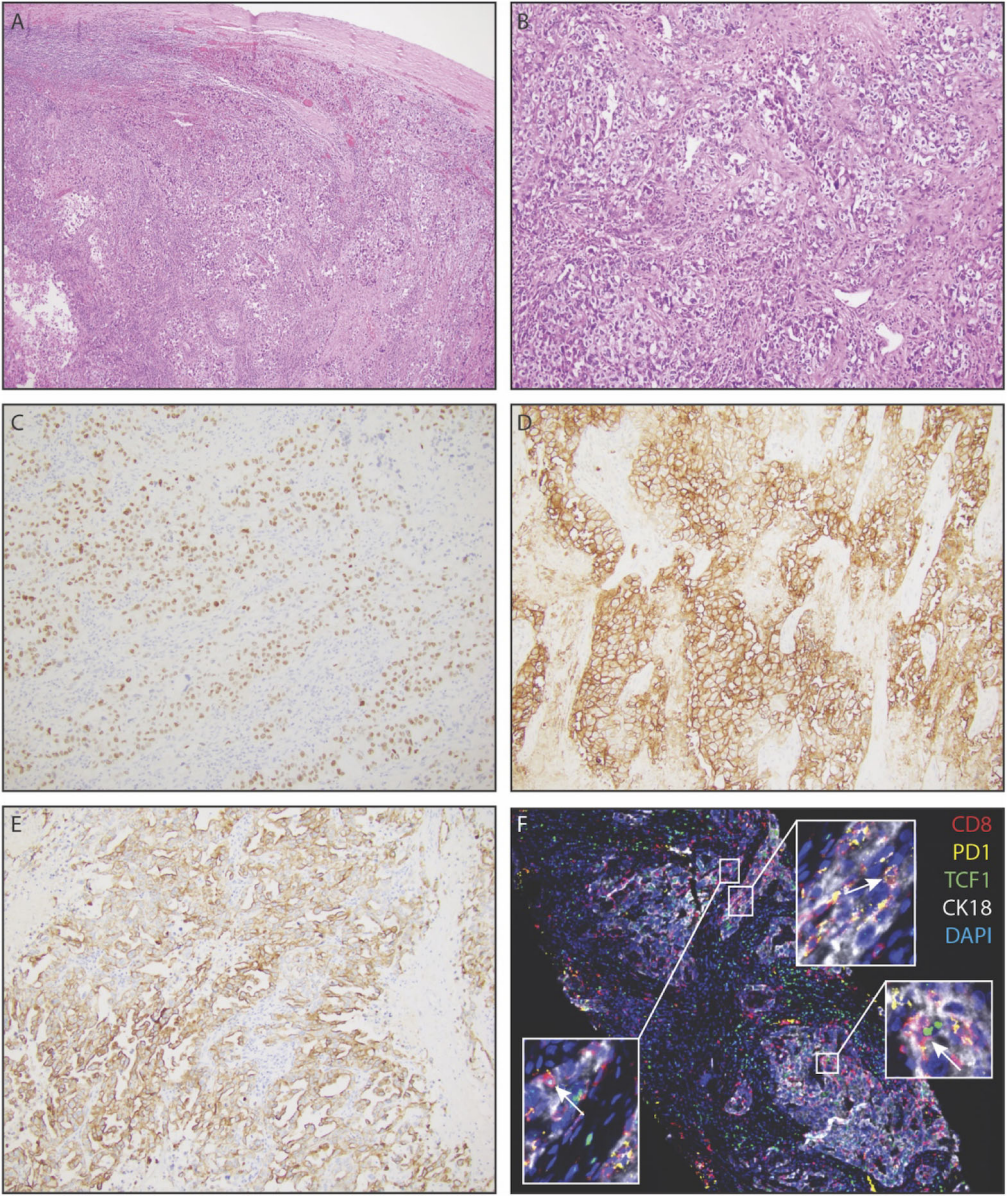
Representative histopathology of extrarenal renal cell carcinoma, demonstrating clear cell renal cell carcinoma in the spleen. **(A)** Low power view of renal cell carcinoma within splenic parenchyma. **(B)** Solid nests of renal cell carcinoma with partially clear cytoplasm in a fibrotic stroma. **(C)** PAX-8 positivity of tumor cells, a pan real marker. **(D)** Carbonic anhydrase IX (CAIX) positivity of tumor cells. **(E)** Diffuse positivity for cytokeratin 7 amongst tumor cells. **(F)** Immunofluorescence staining demonstrating CK18+ tumor cells with infiltrating PD1+CD8+T- cells and PD1+TCF1+CD8+T-cells (white arrows).

The patient was then referred to an academic center in December of 2020. At this time, additional staining of the pathology specimen displayed a morphologic and immunohistochemical profile consistent with a renal primary (**Figures 2A, B**), including positive detection of PAX8 and CK7 while being negative for CK20 (**Figures 2C–E**). The cells consisted of anaplastic-looking epithelioid cells in a background of lymphoid tissue, displaying pronounced nuclear pleomorphism and atypical mitotic figures, without sarcomatoid features (**Figure 2**). The case was presented at Tumor Board for a multidisciplinary consultation with urology, radiation oncology, and pathology, and the consensus analysis determined this presentation to be most consistent with eccRCC. Repeat imaging revealed a new soft tissue nodule adjacent to the posterior pancreatic tail and fundus of the stomach, as well as redemonstrated the peripancreatic fluid collection and the deep, left hemipelvic soft tissue mass (**Figure 1C**). No suspicious lesions were identified in the kidneys. Biopsy of the pelvic mass confirmed metastatic disease. Experimental (non-clinical) immunofluorescence staining was performed on the last biopsy specimen, for infiltrating immune cells, which revealed PD1+ CD8+T cells surrounding and infiltrating nests of CK18+ tumor cells (**Figure 2F**). Notably, this staining demonstrated PD1+ TCF1+ CD8+T cells, which may likely represent the “stem-like” CD8+T cells previously described in RCC (18, 19).

At this time, Foundation One testing of the primary tumor revealed a tumor mutational burden of 10 mutations per megabase, PDL1 amplification, and PDL2 amplification, as well as microsatellite stability, PTEN loss of exon 2, AURKA amplification, GNAS amplification, JAK2 amplification, 2NF217 amplification, and TP53 loss of exons 2–9. Foundation One PDL1 staining revealed PDL1 positivity at 100%. Given these findings demonstrating significant potential for positive responsiveness to immune-checkpoint-based therapy, dual immunotherapy (combination anti-PD1 or anti-PDL1 with anti-CTLA4) or combination immunotherapy + VEGF-TKI therapies were discussed as options for first-line therapy. While the patient’s Sjogren’s disease had not ever required immunosuppression, due to this diagnosis, IO/VEGF-TKI (nivolumab + cabozantinib) was selected as the first-line treatment.

In March 2021, the patient began treatment with nivolumab 480 mg IV every 4 weeks and cabozantinib 40 mg PO daily. In May 2021, imaging demonstrated a partial response to therapy with resolution of the peri-pancreatic nodule and a reduction in the soft tissue mass in the pelvis (**Figure 1D**). Owing to cabozantinib-related hand–foot skin reaction (HFSR, or palmar–plantar erythrodysesthesia), the patient required cabozantinib dose reduction during the treatment course. Ultimately, the disease remained stable on cabozantinib 20 mg PO every other day (**Figure 1A**). In February 2023, due to elevations in liver function testing (not requiring steroid administration) and a nearly 2-year duration of therapy (20–22), shared decision-making resulted in discontinuation of therapy and transition to active surveillance. In May 2023, the most recent imaging redemonstrated stable disease, maintaining partial response, and at the time of this report, the patient is alive and well (**Figure 1E**).

## Discussion and conclusions

In this report, we present a case of an adult with eccRCC. This patient’s cancer was determined to be clear cell (renal cell carcinoma-like) histology, though arising in the spleen. The patient first presented to our center after splenectomy, due to the presence of metastatic disease in the pelvis. At that time, the patient had been on combination systemic therapy (nivolumab and cabozantinib) and displayed partial response on this IO/VEGF-TKI combination therapy. Although combination therapy including two immunotherapies, such as nivolumab and ipilimumab, was also considered, the patient’s history of autoimmune disease (Sjogren’s) suggested immunotherapy plus targeted therapy to be a safer option that would reduce the potential risk of irAEs. Furthermore, the choice of combination IO/VEGF-TKI therapy was supported by a recent case publication describing mRCC without an identified primary (though notably not defined as eccRCC) with clinical benefit following administration of IO/VEGF-TKI therapy (23).

As introduced above, primary eccRCC is a very rare disease, and extraordinarily more so in the setting of normal renal anatomy and function, with only few cases reported in literature (3). While standard-of-care treatment or RCC has evolved in the past few years through various clinical trials involving immunotherapy and targeted therapy options (4, 24–27), standard of care for eccRCC has not yet been established due to the rarity of the disease and the localized nature of eccRCC disease typically reported in the literature. Since the majority of cases currently described in literature are localized, they are typically resolved through surgical resection without recurrence in the surveillance period, and there are no reports to our knowledge of localized eccRCC patients receiving neoadjuvant or adjuvant systemic therapy.

While first-line standard-of-care options for metastatic RCC include IO/IO and IO/VEGF-TKI combination therapies (e.g., nivolumab + ipilimumab and nivolumab + cabozantinib, respectively) (28), prognostic data on systemic therapy for eccRCC presenting as metastatic disease or recurrence after surgical resection are also very limited. Thus, systemic therapy for the treatment of eccRCC in general is relatively uncharted waters and is a highly unexplored and underreported area of research (8–14). There are only reports in the literature of patients undergoing systematic therapy, which are discussed below. However, notably, neither publication reports patient response, as in these cases, the patient discontinued therapy at the institution or unfortunately expired quite rapidly (16, 17).

The first case reports an adult male patient who presented with left-sided neck swelling (16). Clinical examination and imaging revealed a left supraclavicular lymph node mass (measuring 4 cm × 3 cm) and a para-aortic mass displacing the left renal vein (measuring 6 cm × 6 cm). After considering the histology and immunohistochemical results, the final diagnosis was intermediate-risk papillary RCC type II with metastasis. Given that neither kidney had evidence of mass lesion or pathology, these findings were concerning for extrarenal RCC. As there was evidence of metastatic disease given distant sites affected at the time of presentation, systemic treatment was elected rather than surgical resection/localized treatment. The patient was initiated on TKIs (due to financial constraints prohibiting utilization of immunotherapy), and response to this regimen was not reported due to discontinuation of therapy at the reporting institution (16).

The second case reports an adult, who presented with fatigue, weakness, and weight loss (17). Physical examination and imaging revealed a left upper quadrant mass near the left adrenal gland, without renal involvement. Laparoscopic resection of the mass and subsequent pathology evaluation revealed grade 3 clear cell RCC. Six weeks after surgical resection, imaging revealed metastasis to the left kidney hilar lymph nodes and the lungs, and the patient was initiated on chemotherapy for systemic treatment. In this case, the patient expired 3 months after surgery due to multi-organ failure, which occurred after the second dose of chemotherapy (specific agent was not reported) (17).

As such, the only two reported cases on systemic therapies (3) do not involve immunotherapy, despite immunotherapy now constituting typical first-line therapy for mRCC, and (4) do not include any notable data on the response to the treatment. Thus, to our knowledge, this case represents the first case of eccRCC (3) treated with immunotherapy and (4) reporting therapeutic response following administration of systemic therapy.

In light of this therapeutic response, we were particularly interested to see that our immunofluorescence imaging revealed PD1+CD8+T cells in the tumor, including those that are TCF1+ and may likely represent the stem-like CD8+T cells our group has previously defined in RCC (**Figure 2**) (18, 19). In a cohort of unselected RCC patients receiving immune checkpoint blockade, we previously showed that the presence of these stem-like CD8+T cells in densely clustered immune niches was associated with clinical benefit following immune checkpoint blockade (19), and several others have demonstrated the mechanistic importance of these stem-like CD8+T cells in the response to immunotherapy in other tumor types (29–33). Accordingly, though this is a single-patient case report, the presence of these PD1+TCF1+CD8+T cells may suggest that the mechanisms of the response to immunotherapy seen in traditional RCC may also apply to eccRCC.

As consensus guidelines or literature suggesting standard-of-care treatment options for eccRCC are lacking, the treatment plan reported in this case was selected by extrapolating standard-of-care IO/VEGF-TKI treatment approaches commonly used in mRCC cases. We show here that this IO/VEGF-TKI combination therapy could be an effective treatment option for treating eccRCC, as evidenced by this patient’s partial response, and may potentially also share mechanisms of response, given the immune infiltration revealed by immunofluorescence staining. Accordingly, this novel approach—treating an eccRCC patient with IO/VEGF-TKI therapy—serves as an important data point for working towards the establishment of a standard-of-care treatment plan for patients with eccRCC in the future.

In summary, here we present a case of a patient with eccRCC, originating in the spleen, which was resected via laparoscopic splenectomy and later recurred and was treated with IO/VEGF-TKI. While additional reports and definitive clinical trials of eccRCC patients treated with IO/IO or IO/VEGF combinations are needed to define optimal treatment strategies, this case alone serves as a foundation example, demonstrating how IO/VEGF treatment regimens (extrapolated from mRCC standard-of-care systemic therapy) could serve as a promising option for eccRCC. Future investigations should give careful attention to identifying eccRCC and work towards establishment of standard-of-care regimens for this rare subtype of disease.

## Data availability statement

The original contributions presented in the study are included in the article/supplementary material. Further inquiries can be directed to the corresponding author.

## Ethics statement

Ethical approval was not required for the study involving humans in accordance with the local legislation and institutional requirements. Written informed consent to participate in this study was not required from the participants or the participants’ legal guardians/next of kin in accordance with the national legislation and the institutional requirements. Written informed consent was obtained from the individual(s) for the publication of any potentially identifiable images or data included in this article. Written informed consent was obtained from the participant/patient(s) for the publication of this case report.

## Author contributions

CJ: Conceptualization, Data curation, Formal Analysis, Methodology, Supervision, Visualization, Writing – original draft, Writing – review & editing. YC: Data curation, Investigation, Project administration, Writing – original draft, Writing – review & editing. SE: Conceptualization, Supervision, Writing – original draft, Writing – review & editing. RG: Data curation, Methodology, Writing – review & editing. JB: Data curation, Supervision, Writing – review & editing. CH: Conceptualization, Supervision, Writing – review & editing. HK: Conceptualization, Resources, Supervision, Writing – review & editing. LH: Data curation, Investigation, Methodology, Resources, Supervision, Visualization, Writing – review & editing. MB: Conceptualization, Data curation, Formal Analysis, Funding acquisition, Investigation, Project administration, Resources, Supervision, Writing – original draft, Writing – review & editing.
